# What specific exercise training is most effective exercise training method for patients on maintenance hemodialysis with sarcopenia: a network meta-analysis

**DOI:** 10.3389/fnut.2024.1484662

**Published:** 2024-11-22

**Authors:** Ying Li, Jingjing Li, Xiaoan Chen, Yuegong Shi, Jie Shen, Ting Huang

**Affiliations:** ^1^College of Sports Science, Jishou University, Jishou, China; ^2^Blood Purification Center, The Fourth People’s Hospital of Lianyungang, Affiliated Hospital of Nanjing Medical University Kangda College, Jiangsu, China; ^3^Department of Nephrology, Shanghai East Hospital, Tongji University School of Medicine, Shanghai, China; ^4^Nursing Department, The Third People’s Hospital of Ganzhou, Ganzhou, Jiangxi, China

**Keywords:** MHD, maintenance hemodialysis, sarcopenia, network meta-analysis, meta

## Abstract

**Objective:**

The present study aimed to investigate the influence of different exercise methods on sarcopenia patients receiving maintenance hemodialysis (MHD) by conducting a network meta-analysis.

**Methods:**

The PubMed, Embase, Cochrane Library, Web of Science, China National Knowledge Infrastructure (CNKI), and Wanfang databases were searched online for relevant articles published until May 2024. Based on the inclusion and exclusion criteria, we selected 10 articles that compared the effects of 7 exercise interventions on sarcopenia patients receiving MHD.

**Results:**

The results of network meta-analysis showed that resistance training (RT) [standardized mean difference (SMD) = 4.54; 95% confidence interval (CI): 3.27–5.80] significantly improved the handgrip strength (HGS) of sarcopenia patients receiving MHD as compared to Baduanjin exercise (BAE) (SMD = 4.19; 95% CI: 2.31–6.07), bicycle exercise (BIE) (SMD = 4.06; 95% CI: 0.02–8.10), and combined movement (CE) (SMD = 3.50; 95% CI: 3.13–3.87). Compared to normal care (NC), BAE (SMD = 0.15; 95% CI: 0.07–0.23), RT (SMD = 0.34; 95% CI: 0.06–0.62), and CE (SMD = 0.37; 95% CI: 0.16–0.58) significantly improved skeletal muscle mass index (SMI) in sarcopenia patients receiving MHD.

**Conclusion:**

This study showed that RT has a positive effect on improving HGS in sarcopenia patients receiving MHD. CE also showed good results in enhancing SMI in MHD patients with sarcopenia. More randomized controlled trials are required to better understand the effectiveness of these exercise interventions and the potential underlying mechanisms.

## Introduction

Patients with end-stage renal disease (ESRD) require kidney replacement therapy such as dialysis or transplantation for their survival. Maintenance hemodialysis (MHD) is the most widely used dialysis method for ESRD patients. However, patients receiving hemodialysis have multiple catabolic issues.

In patients with chronic kidney disease (CKD), protein energy wasting (PEW) due to decreased anabolism and increased catabolism results in the development of sarcopenia. However, PEW is not the sole factor for sarcopenia development and is not characterized only by deficiencies in protein metabolism. PEW occurs in 15–74% of patients receiving MHD (1, 2). PEW reduces muscle protein synthesis in skeletal muscle and increases the rate of muscle proteolysis, leading to sarcopenia (3). The onset of sarcopenia, characterized by muscle weakness, decreased muscle function, low exercise capacity, and reduced physical activity, begins in the early stages of CKD and progresses with ESRD (4–6). According to previous studies, the prevalence of sarcopenia in MHD patients is 18% (7). A meta-analysis by Li et al. (8) revealed that the prevalence of sarcopenia was 33 and 32% in Asian and Chinese patients receiving MHD, respectively. Pharmacological, dietary, and exercise interventions are also used for treating PEW and sarcopenia in patients receiving MHD. However, to date, there are no effective drugs for treating PEW and sarcopenia in patients on MHD. Furthermore, patients with MHD often experience fatigue and lack of energy. As reported previously, patients with MHD can well tolerate appropriate physical exercise; thus, exercise intervention could be an effective strategy to improve symptoms in patients on MHD (3). Several studies have shown that exercise intervention can effectively delay the occurrence of sarcopenia, improve patients’ quality of life, and prolong their survival; thus, it is currently the most effective measure to treat sarcopenia (9, 10). Resistance exercise (RE) refers to exercises that improve the strength, endurance, and size of skeletal muscles (11). Li et al. (12) conducted a traditional meta-analysis and found that RE improved grip strength and muscle mass in sarcopenia patients receiving MHD. Aerobic exercise (AE) such as walking, cycling, and jogging in any land-based mode that is designed to improve the efficiency and capacity of the cardiorespiratory system (11). Meng et al. (13) found that AE and RE can improve grip strength and serum albumin levels in sarcopenia patients receiving MHD. However, because exercise interventions are diverse in nature and have distinctive characteristics, a consensus is required to determine the most effective exercise interventions.

Network meta-analysis (NMA), or meta-analysis of studies reporting mixed treatment or comparison of multiple treatments (14), can be used to compare the effectiveness of different exercise interventions in sarcopenia patients on MHD through direct and indirect comparisons. Currently, sarcopenia is a hot topic of research at the international level. Although studies on the effects of different exercise methods on sarcopenia have been reported, further NMA-based studies are required on the comparison of various exercise methods on sarcopenia patients receiving MHD. Therefore, the present study aimed to compare the effects of different exercise interventions on sarcopenia patients receiving MHD by conducting NMA of relevant randomized controlled trials. The results of this study could contribute to develop clinical practice guidelines to recommend the most effective exercise interventions to advise and guide exercise choices for sarcopenia patients receiving MHD.

## Methods

This NMA was designed based on the guidelines for Preferred Reporting Items of Systematic Review and Network Meta-Analysis (15) and registered in the PROSPERO database (CRD42024575389).

### Selection of research articles

A literature search of the PubMed, Embase, Cochrane Library, Web of Science, China National Knowledge Infrastructure (CNKI), and Wanfang databases was conducted by YL and YS, and the titles and abstracts of the retrieved literature were screened using the previously established search strategy to identify relevant studies that met the inclusion criteria. The search strategy utilized a combination of Mesh Terms and free-text terms. Can be accessed in Appendix 1. In the case of disagreements, a third author (CX) was consulted to reach a consensus. EndNote software was used to remove duplicate records (16) to ensure data integrity. Full-text articles from potentially eligible studies were evaluated thoroughly based on the predetermined inclusion and exclusion criteria.

### Inclusion and exclusion criteria

The inclusion and exclusion criteria were based on PICOS standards (Table 1).

**Table 1:** Inclusion and exclusion criteria.

Category	Inclusion criteria	Exclusion criteria
Population	Sarcopenia patients receiving MHD (dialysis duration >3 months)	Patients with severe high blood pressure, heart disease, or other severe systemic diseases
Interventions	Home exercise (HE), resistance training (RT), stretching exercise (SE), combined movement (CE), bicycle exercise (BIE), Baduanjin exercise (BAE)	
Comparisons	Normal care (NC)	
Outcomes	Handgrip strength (HGS), skeletal muscle mass index (SMI)	
Study	Randomized controlled trial; published in English or Chinese	Non-full-text and republished literature; studies whose data cannot be obtained or transformed

All interventions and outcome measures can be accessed in Appendices 2, 3.

### Data extraction

The authors independently extracted the following data from the included studies: first author, year of publication, country of origin, sample size, duration of dialysis, age, intervention duration, intervention frequency, and outcome measures.

### Risk of bias assessment

The risk of bias was independently assessed by two reviewers and resolved by a third reviewer by using Cochrane Collaboration’s tools (17), including sequence generation, blinding, incomplete data, nonselective reporting of results, and other sources of bias. Each criterion was assessed to have a low, unclear, or high risk of bias.

### Data analysis

Review Manager 5.3 was used to plot risk bias and perform the NMA. Stata 15.0 was used for grid element analysis with the consistency model. For the various exercise interventions, all outcome measures were considered continuous variables and expressed as mean, standard deviation (SD), and mean-variance (MD), which represent the absolute difference between the treatment and control groups and is calculated using the same sample size, or standardized mean difference (SMD), which is obtained by dividing the mean value of the groups by the standard deviation between subjects and is suitable for data analysis in trials of varying sample sizes, along with 95% confidence interval (CI) (18). The network plot function was used to generate network diagrams to present the different forms of intervention modes. We used nodes to represent various interventions and edges to illustrate comparisons between these interventions. Node splitting tests assessed local inconsistencies between direct and indirect evidence. The difference between the direct and indirect coefficients, calculated as the *p*-value, was used to detect inconsistencies. A *p*-value of <0.05 indicated a local inconsistency, wherein non-transitivity was suspected, and potential influencing factors were examined (19). The effects of different exercise interventions on handgrip strength (HGS) and skeletal muscle mass index (SMI) in sarcopenia patients receiving MHD were estimated according to the Surface Under the Cumulative Ranking Curve (SUCRA). Efficacy ranking was determined with Stata, and cumulative probability ranking was plotted to obtain SUCRA (20). The higher the SUCRA value, the higher was the probability of being considered the most effective intervention (21). While determining SUCRA rankings, in addition to comparison of the area under the curve value for the SUCRA plot of cumulative ranking probability of different exercise interventions, careful interpretation of the clinical significance of these interventions is required. Furthermore, to address the possibility of publication bias in the NMA, we constructed a network funnel plot and visually evaluated its symmetry to detect the presence of small sample effects (21).

## Results

### Literature screening process

**Figure 1:**
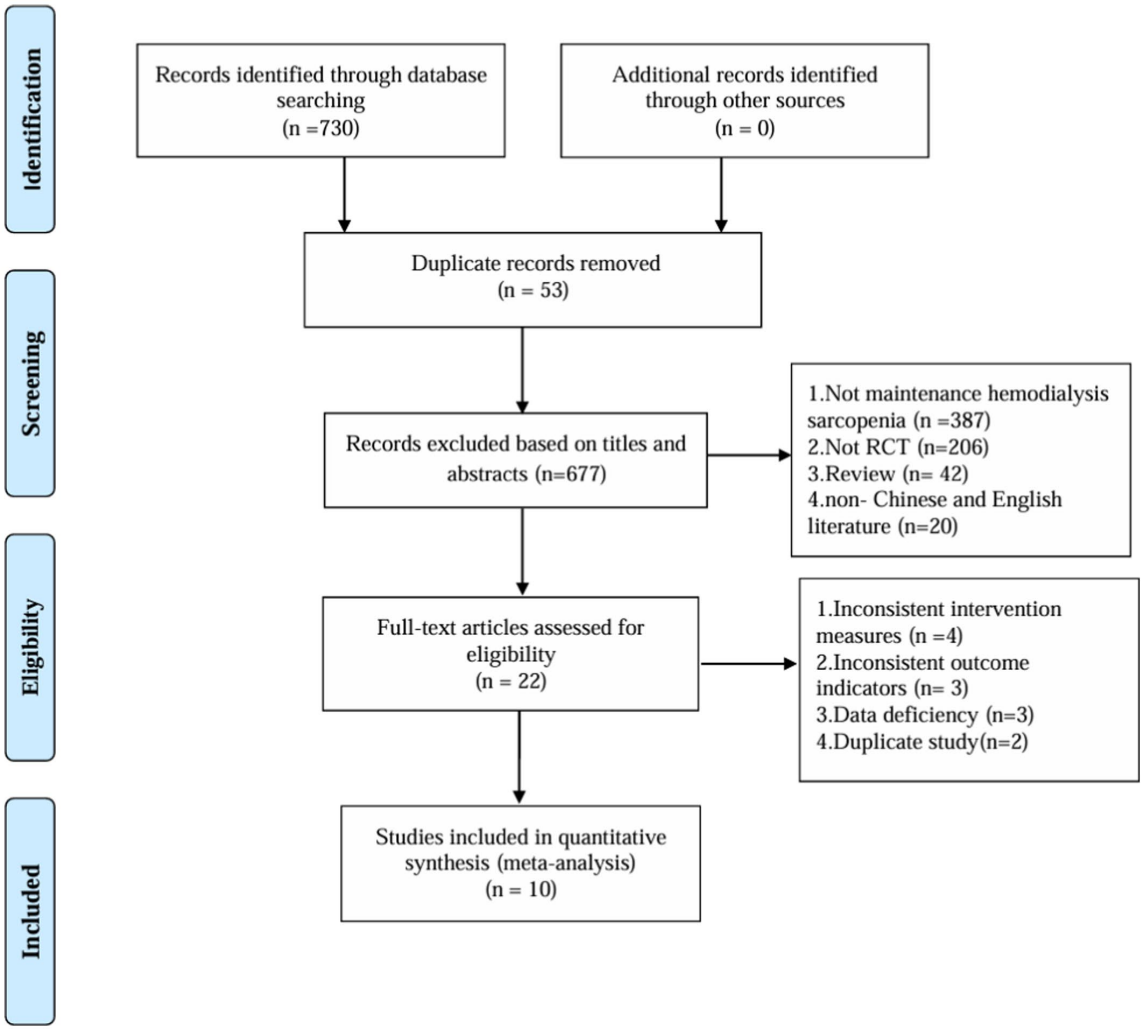
Flowchart of the article selection process.

After a thorough search across multiple databases, the initial screening process identified 730 relevant articles. A total of 53 duplicate entries were removed using Endnote software. Subsequently, 655 articles were excluded based on their titles and abstracts. The remaining 22 articles were subjected to full-text evaluation, and finally, 10 articles were selected for the NMA (Figure 1).

### Study and patient characteristics

**Figure 2:**
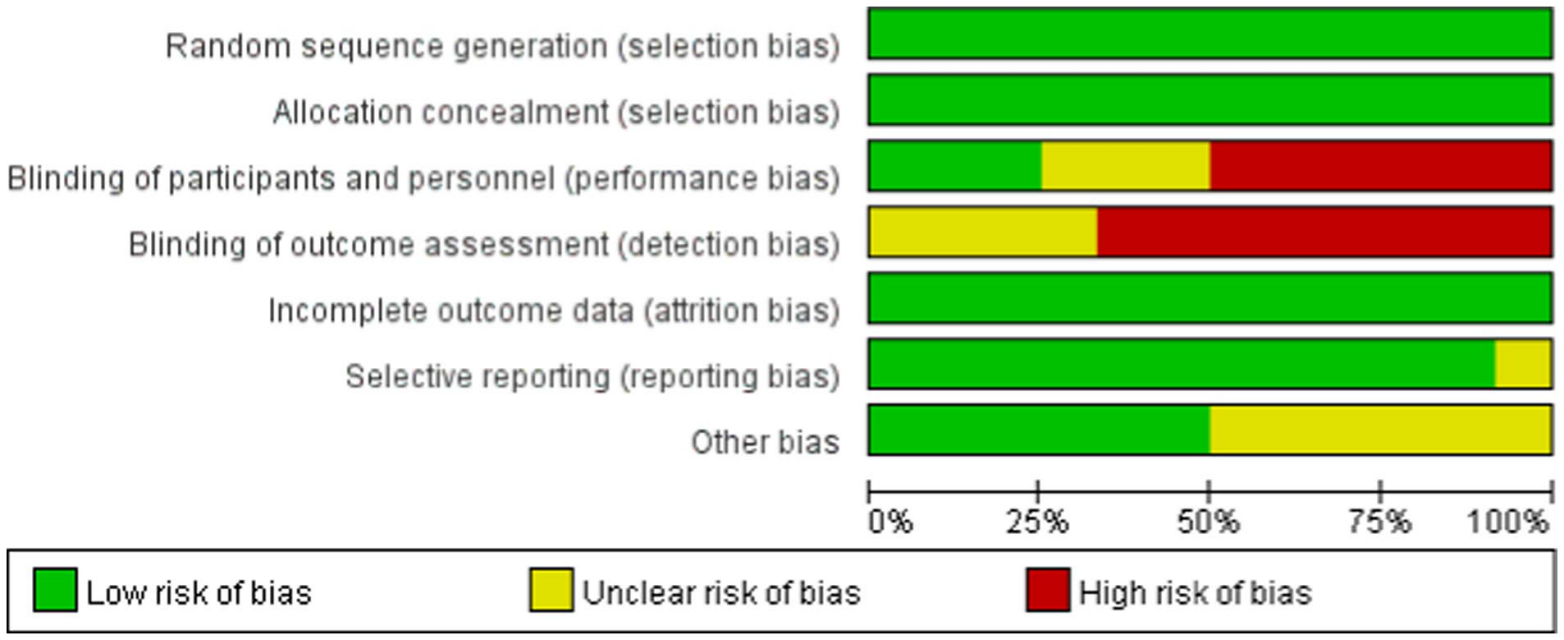
Percentages of the included studies with low, unclear, and high risk of bias based on the features of the Cochrane bias risk tool.

Our NMA included studies that compared the effects of seven different exercise interventions on sarcopenia patients receiving MHD and were published between 2012 and 2023. The included studies comprised 589 patients, with an intervention duration of 8–12 weeks. HGS and SMI were reported in 12 and 5 studies, respectively. The average age of the patients was 52–73 years, with a mean BMI of 18–29 and dialysis duration of 1.54 (years) to 80.97 (months). Table 2 shows the characteristics of the studies and patients. The risk assessment of bias for each study is shown in Figure 2.

**Table 2:** General characteristics of the included studies.

Name	Years	Country	Type of exercise	Age	BMI	Male/female	Duration of dialysis (month)	Intervention time	Intervention frequency	Outcomes
Dong (37)	2019	China	RT/NC	59.0 (32.5, 66.5)/62.5 (50.5, 70.0)	18.96 ± 3.08/20.49 ± 3.41	RT: 9/12 NC: 12/8	RT: 69.0 (31.5, 87.5) NC: 57.5 (32.5, 86.5)	12 weeks	3 times a week	HGS; SMI
Wang (38)	2023	China	RT/NC	59.45 ± 5.54/59.32 ± 5.21	NA	RT: 21/17 NC: 22/16	RT: 19.81 ± 6.64 NC: 19.35 ± 6.78	NA	3 times a week	HGS; SMI
Lopes (39)	2019	USA	RT/SE	56.2 ± 12.5/54.6 ± 12.4	26.3 ± 3.7/25.5 ± 5.1	RT: 13/7 SE: 9/7	RT: 72.1 ± 50.3 SE: 53.2 ± 44.1	12 weeks	3 times a week	HGS; SMI
Song (40)	2012	Korea	RT/NC	52.1 ± 12.4/54.6 ± 10.1	NA	RT: 8/12 NC: 12/8	RT: 38.9 ± 26.1 NC: 45.9 ± 56.2	12 weeks	NA	HGS
Zhang (3)	2020	China	RT/NC	60.0 (51.0, 66.0)/62.0 (54.0, 68.0)	22.8 ± 2.94/22.51 ± 3.08	RT: 27/16 NC: 26/18	3RT: 9.0 (23.0, 89.0) NC: 30.5 (19.3, 78.8)	12 weeks	3 times a week	HGS
da Costa Rosa (41)	2018	Brazil	RT/NC	54.49 ± 11.97/57.10 ± 16.20	NA	RT: 20/8 NC: 15/9	RT: 1.54 ± 1.26 years NC: 2.35 ± 1.66 years	12 weeks	NA	HGS
Chan (42)	2019	USA	HE/NC	66.5 ± 7.4/66.3 ± 6.7	28.5 ± 3.3/29.1 ± 4.6	HE: 11/2 NC: 10/5	NA	12 weeks	NA	HGS
Meng (13)	2023	China	CE/NC	73.77 ± 7.3/73.83 ± 6.73	28.27 ± 2.93/28.22 ± 3.41	CE: 15/14 NC: 15/15	CE: 67.25 ± 39.15 NC: 80.97 ± 52.01	12 weeks	NA	HGS; SMI
Zhu (43)	2022	China	BIE/NC	65.32 ± 5.45/68.84 ± 7.44	22.82 ± 3.07/22.83 ± 3.02	BIE: 18/7 NC: 15/15	BIE: 46.64 ± 42.171 NC: 72.68 ± 49.481	12 weeks	3 times a week	HGS
Qian (44)	2021	China	BAE/NC	57.12 ± 1.56/56.98 ± 10.67	NA	BAE: 30/28 NC: 30/27	BAE: 77.66 NC: 78.7	12 weeks	3 times a week	HGS; SMI

HE, home exercise; RT, resistance training; SE, stretching exercise; CE, combined movement; BIE, bicycle exercise; BAE, Baduanjin exercise; NC, normal care. Data are expressed as mean ± standard deviation.

### NMA outcomes

#### HGS

**Figure 3:**
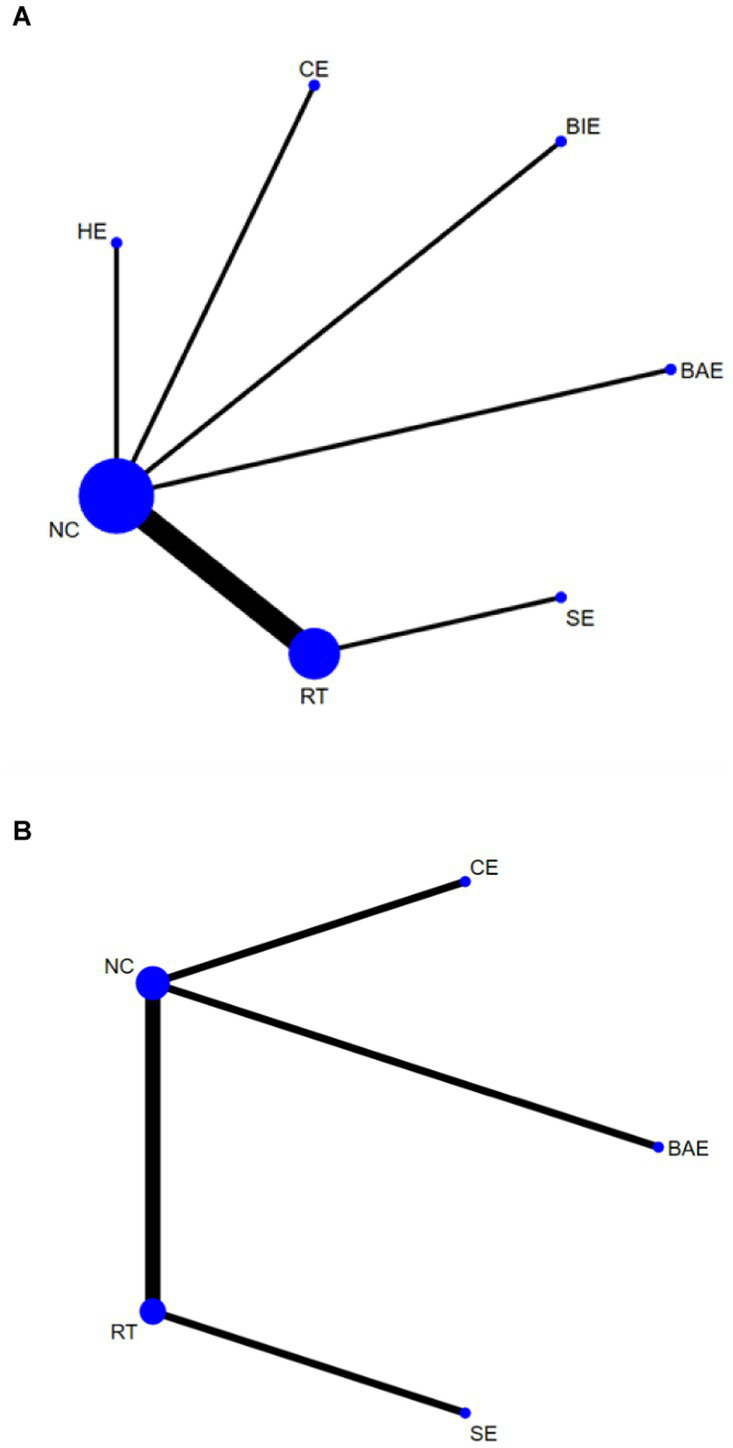
Network plots: **(A)** HGS, **(B)** SMI. The size of the nodes represents the number of times the exercise appears in any comparison of that treatment, and the width of the edges represents the total sample size in the comparisons it connects. HE, home exercise; RT, resistance training; SE, stretching exercise; CE, combined movement; BIE, bicycle exercise; BAE, Baduanjin exercise; NC, normal care.

**Figure 4:**
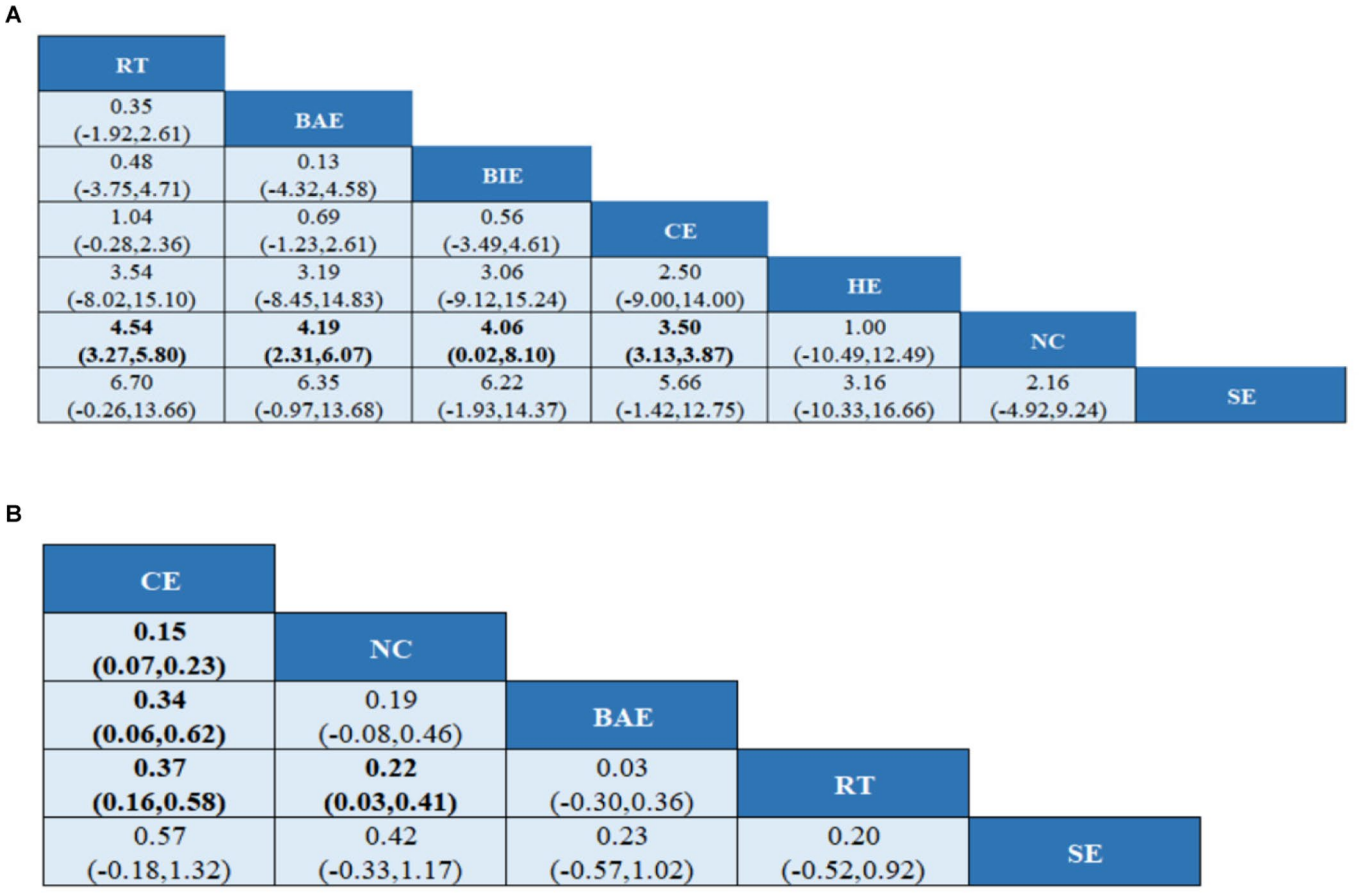
League tables of outcome analyses **(A)** HGS, **(B)** SMI. HE, home exercise; RT, resistance training; SE, stretching exercise; CE, combined movement; BIE, bicycle exercise; BAE, Baduanjin exercise; NC, normal care. Data are expressed as mean difference and 95% CI for continuous data.

**Figure 5:**
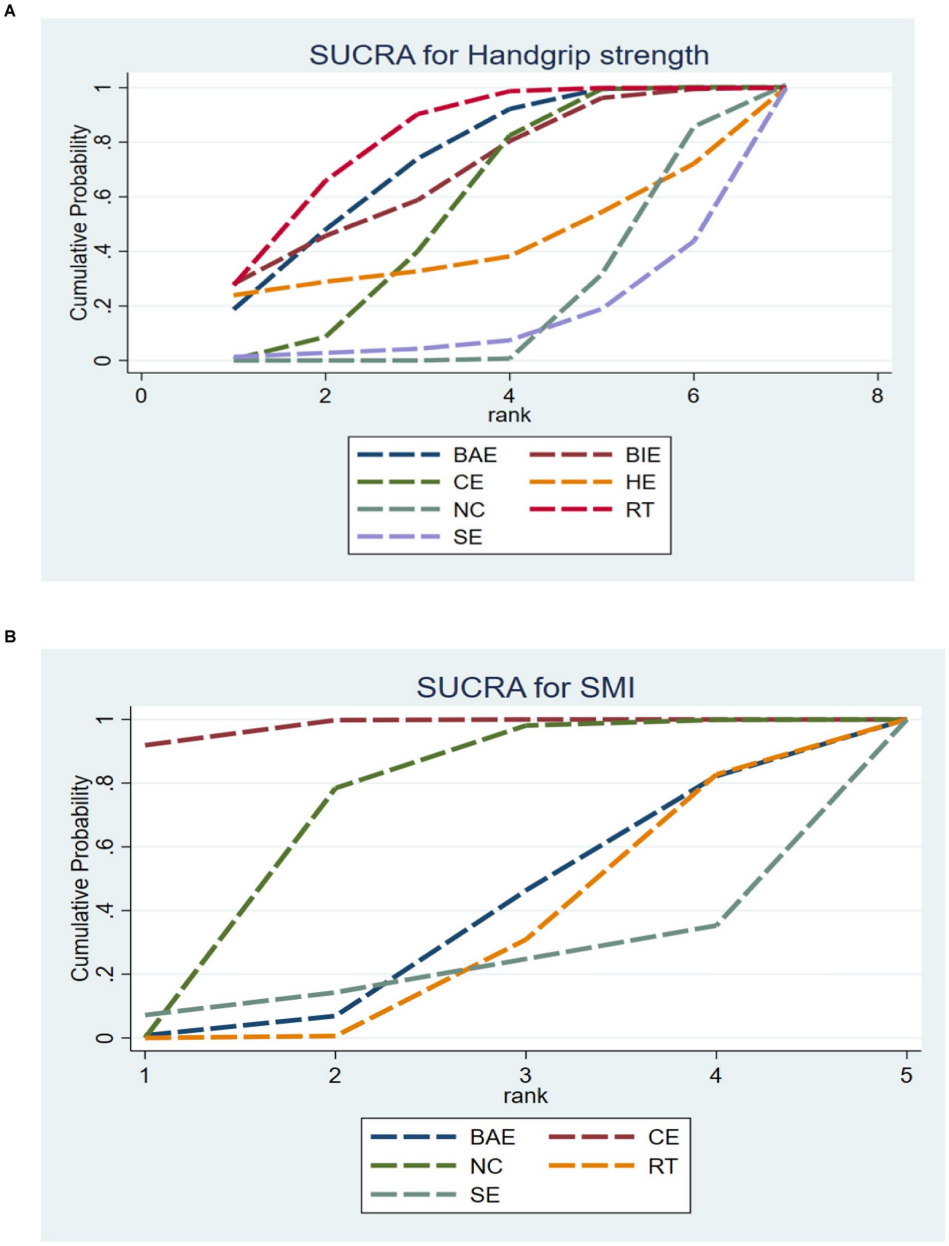
SUCRA plot of **(A)** HGS and **(B)** SMI. HE, home exercise; RT, resistance training; SE, stretching exercise; CE, combined movement; BIE, bicycle exercise; BAE, Baduanjin exercise; NC, normal care.

**Figure 6:**
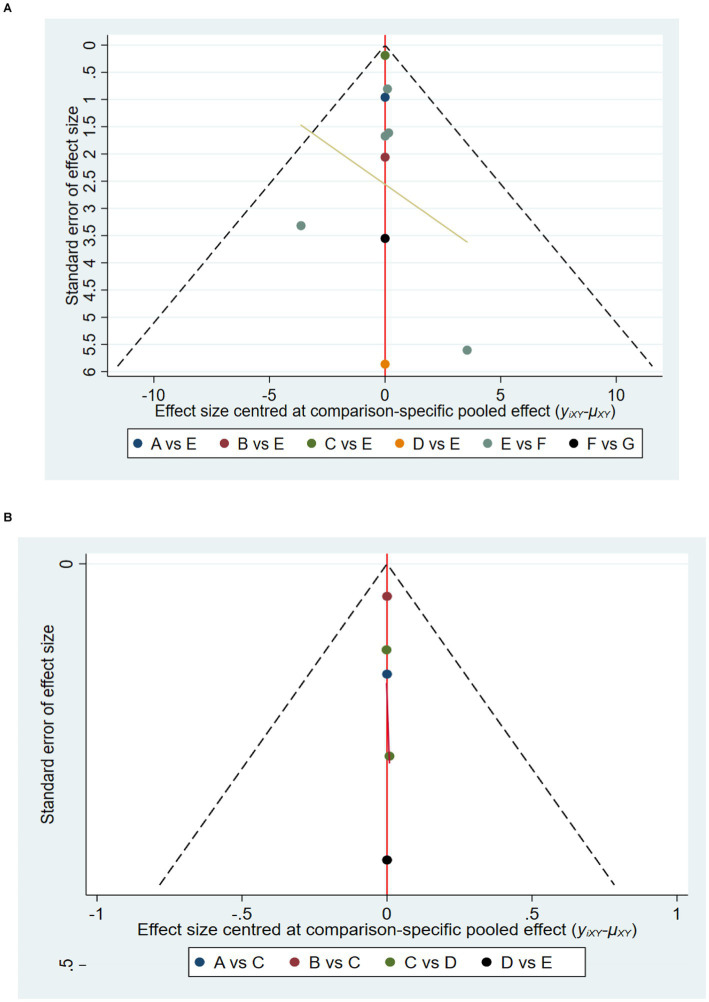
Funnel chart: **(A)** HGS and **(B)** SMI. A = BAE, Baduanjin exercise; B = BIE, bicycle exercise; C = CE, combined movement; D = HE, home exercise; E = NC normal care; F = RT, resistance training; G = SE, stretching exercise.

A total of 10 studies involving 589 participants assessed HGS. In the NMA, 7 interventions were included (Figure 3A): home exercise (HE), resistance training (RT), stretching exercise (SE), combined movement (CE), bicycle exercise (BIE), Baduanjin exercise (BAE) and normal care (NC). RT significantly improved the HGS of MHD patients with sarcopenia compared with BAE, BIE and CE (SMD = 4.54; 95% CI: 3.27–5.80), (SMD = 4.19, 95% CI: 2.31–6.07), (SMD = 4.06, 95% CI: 0.02–8.10), (SMD = 3.50, 95% CI: 3.13–3.87) (Figure 4A). In addition, direct comparisons of HGS were evaluated (Appendix 4.1). Figure 5A shows SUCRA for all modes of exercise. SUCRA is able to predict the likelihood of different movements and serve as a reference for selecting the best treatment. The results showed that RT was most likely to be the best exercise measure (81.0%). The others are BAE (71.9%), BIE (68.3%), CE (54.9%), HE (41.0%), NC (19.8%), SE (13.1%), and the funnel diagram is shown in Figure 6A.

#### SMI

A total of 5 studies involving 327 participants evaluated HGS. In the NMA, 5 interventions were included (Figure 3B): stretching exercise (SE), combined movement (CE), resistance training (RT), Baduanjin exercise (BAE) and normal care (NC). Compared with NC, BAE, and RT, CE significantly improved SMI in MHD patients with sarcopenia (SMD = 0.15; 95% CI: 0.07–0.23), (SMD = 0.34; 95% CI: 0.06–0.62), (SMD = 0.37, 95% CI: 0.16–0.58) (Figure 4B). In addition, a direct comparison of SMI was evaluated (Appendix 4.2). Figure 5B shows the SUCRA for all motion modes. SUCRA can predict the likelihood of different movements and serve as a reference for selecting the best treatment. The results showed that CE was most likely the best exercise measure (98.0%). The others were NC (69.0%), BAE (35.0%), RT (28.4%) and SE (19.6%), as shown in Figure 6B.

## Discussion

NMA methods have been used to determine the optimal type or intensity of exercise for the rehabilitation of patients with some chronic diseases (22, 23). However, thus far, very few NMAs have examined exercise patterns in sarcopenia patients with hemodialysis. Therefore, in the present study, we conducted an NMA to explore the optimal exercise mode for sarcopenia patients receiving MHD. The results showed some interesting findings, and we recommend that resistance training (RT) and combined movement (CE) are the best types of exercise to improve HGS and SMI in sarcopenia patients on MHD.

Sarcopenia is a skeletal muscle syndrome characterized by reduced muscle mass, muscle strength, and muscle function (24). HGS is an indicator of skeletal muscle strength, which can directly reflect not only the muscle strength of the hand but also the total strength of the muscles in other parts of the body (25). Based on the NMA, the present study found that RT is the best exercise to improve grip strength in sarcopenia patients with MHD. RT enhances muscle function by increasing the cross-sectional area of muscle fibers and muscle mass (26). Previous studies have shown that RT involves an active movement of muscles against external resistance, which can increase the cross-sectional area of type I and type II muscle fibers in older people. Hence, the intervention results are ideal for the symptoms of reduction in the cross-sectional area of muscle fiber, muscle strength, and contractile function in patients with sarcopenia (13). RT can stimulate muscle growth by increasing oxygen consumption in patients on MHD (27), which can protect muscle mass and improve exercise capacity in patients receiving MHD, thereby improving muscle strength in these patients. Although RT has a good effect on improving muscle strength in sarcopenia patients with MHD, the conditions of self-tolerance and dialysis should also be considered in RT for sarcopenia patients with MHD, and attention should be paid to the individualized adjustment of exercise intensity and protection of limbs on the arteriovenous fistula side during exercise. However, because our study did not compare exercise intensity and exercise frequency, the appropriate exercise intensity and exercise frequency should be selected according to the tolerance and clinical conditions of patients on MHD in clinical practice. To avoid the high risk of exercise for patients, a multidisciplinary team should use a staging model to customize a resistance exercise plan and conduct a dynamic assessment of the exercise conditions of patients on MHD to achieve the expected exercise goals while ensuring patient safety (28).

SMI is an important indicator to evaluate sarcopenia. CE refers to the use of two or more specific types of exercise training (29). In the present study, CE (RT + AE) showed the best effect in improving SMI in sarcopenia patients receiving MHD. Aerobic exercise (AE), also known as “cardiopulmonary function training,” provides the energy required for exercise through aerobic metabolism (30). Aerobic exercise involves repeated exercise of many muscle groups, which increases the energy production of mitochondria and improves oxygen uptake efficiency and muscle endurance. There is a simultaneous increase in the capacity of muscle capillaries to meet the requirements of muscle mitochondria for increased oxygen uptake (31). AE can also increase the number of mitochondria in skeletal muscle cells, particularly in aging skeletal muscle cells, by activating multiple transcription factors, promoting the change in myosin heavy chain from a rapid change to a gradual change, and ultimately inhibiting the degradation of skeletal muscle protein, thereby improving body metabolism and reducing the proportion of body fat. Furthermore, aerobic exercise increases fat-free weight and muscle mass, reduces chronic inflammation, minimizes risk factors for metabolic diseases, and improves cardiorespiratory function and mobility (32). RT also stimulates vasodilation; delivers nutrients to muscles; promotes muscle fibrin increase; continuously inhibits muscle breakdown throughout the body; induces changes in the level of assimilating hormones such as auxin; and inhibits the production of myostatin to improve muscle quantity, quality, and strength (33). CE can avoid the drawbacks of only one type of exercise. However, some studies have found that additional resistance exercise is one of the factors leading to fatigue in patients (34). In the present study, only one type of CE was included, and further research on CE is required for other sport activities. Future studies should include more literature to clarify the advantages of CE. We also suggest that clinicians or therapists should comprehensively consider the patient’s condition and provide personalized, combined intervention for sarcopenia patients on hemodialysis.

The current intervention methods mainly include exercise, nutrition, and combined interventions to delay the occurrence and development of sarcopenia, reduce its symptoms, and improve patients’ quality of life. Nonpharmacological interventions such as exercise and nutrition were found to be effective in patients on MHD. In addition to exerting a health-promoting effect, these interventions are more economical and convenient and can be applied to a wide range of patients. In the present study, we compared only the impact of different exercise interventions on sarcopenia in patients receiving MHD. We found that the microinflammatory state is an independent predictor of sarcopenia development in patients receiving MHD, and the chronic inflammatory response can promote muscle breakdown and inhibit protein synthesis, resulting in muscle loss (35). Additionally, MHD treatment requires limiting the intake of nutrients such as proteins, sodium, and potassium; not all patients can normally consume adequate nutrients. Therefore, protein metabolism disorder is an essential pathogenetic manifestation of sarcopenia. Hence, scientific and adequate nutritional support is necessary for patients on MHD to prevent and alleviate sarcopenia. The KDOQI Clinical Practice Guideline on CKD Nutrition (2020) recommends that metabolically stable patients on MHD should ensure a dietary protein intake of 1.0 to 1.2 g/kg body weight per day to maintain a stable nutritional status (36). However, it is essential to note that patients on MHD can also have other physical conditions and healthcare needs. The appropriate exercise and nutrition regimen are designed for patients and regularly evaluated and modified to ensure that patients achieve the best results from the exercise intervention for alleviating sarcopenia.

## Conclusion

Evidence from an NMA strongly supports the idea that RT can improve HGS in sarcopenia patients receiving MHD. Furthermore, CE can improve SMI in sarcopenia patients on MHD. The present study, however, yielded limited results, and future studies should incorporate more research to strengthen these results and identify appropriate exercise interventions for sarcopenia patients on MHD.

## Data Availability

The original contributions presented in the study are included in the article/Supplementary material, further inquiries can be directed to the corresponding author.
