# Supplementary material for: What specific exercise training is most effective exercise training method for patients on maintenance hemodialysis with sarcopenia: a network meta-analysis
Source: Front Nutr. 2024 Nov 22;11:1484662. doi: 10.3389/fnut.2024.1484662 (PMC11622696; doi:10.3389/fnut.2024.1484662)
Supplement: Supplementary file 1 [file Data_Sheet_1.ZIP › Supplementary material/Appendix 1-Search strategy.DOCX]

**Pubmed:**

| Number | Search terms | Results |
| --- | --- | --- |
| **#1** | ((((((Hemodialysis [MeSH Terms]) OR (Dialyses, Renal [Title/Abstract])) OR (Renal Dialyses [Title/Abstract])) OR (Dialysis, Renal [Title/Abstract])) OR (Hemodialyses [Title/Abstract])) OR (maintenance hemodialysis [Title/Abstract])) OR (MHD[Title/Abstract]) | 160,456 |
| **#2** | ((Sarcopenia [MeSH Terms]) OR (Sarcopenias [Title/Abstract])) OR (Muscular Atrophy [Title/Abstract]) | 22,011 |
| **#3** | #1 AND #2 | 236 |
| **#4** | ((((((((((((((((((((((((((Exercises[Title/Abstract]) OR (Exercise, Physical[Title/Abstract])) OR (Exercises, Physical[Title/Abstract])) OR (Physical Exercise[Title/Abstract])) OR (Physical Exercises[Title/Abstract])) OR (Physical Activity[Title/Abstract])) OR (Activities, Physical[Title/Abstract])) OR (Activity, Physical[Title/Abstract])) OR (Physical Activities[Title/Abstract])) OR (Exercise, Aerobic[Title/Abstract])) OR (Aerobic Exercise[Title/Abstract])) OR (Aerobic Exercises[Title/Abstract])) OR (Exercises, Aerobic[Title/Abstract])) OR (Training, Resistance[Title/Abstract])) OR (Strength Training[Title/Abstract])) OR (Training, Strength[Title/Abstract])) OR (Weight-Lifting Strengthening Program[Title/Abstract])) OR (Weight Lifting Strengthening Program[Title/Abstract])) OR (Weight-Lifting Strengthening Programs[Title/Abstract])) OR (home based exercise[Title/Abstract])) OR (Exercise Tests[Title/Abstract])) OR (Test, Exercise[Title/Abstract])) OR (Tests, Exercise[Title/Abstract])) OR (Exercise Testing[Title/Abstract])) OR (Testing, Exercise[Title/Abstract])) OR (bicycle exercise[Title/Abstract])) OR (Baduanjin[Title/Abstract]) | 265,562 |
| **#5** | #3 AND #4 | 25 |
