# Supplementary figures and images for: What specific exercise training is most effective exercise training method for patients on maintenance hemodialysis with sarcopenia: a network meta-analysis
Source: Front Nutr. 2024 Nov 22;11:1484662. doi: 10.3389/fnut.2024.1484662 (PMC11622696; doi:10.3389/fnut.2024.1484662)

# Treatment Effect

# Mean with 95%CI

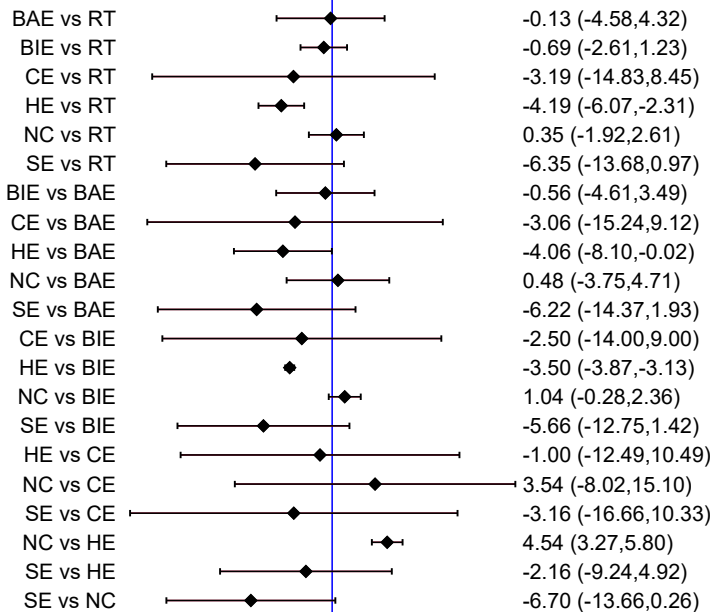

-17 -8.7 0 7.2 15

Supplement: Supplementary file 1 [file Data_Sheet_1.ZIP › Supplementary material/Appendix 4.1-Pairwise comparisons of forest plots.pdf]

## Treatment Effect

## Mean with 95%CI

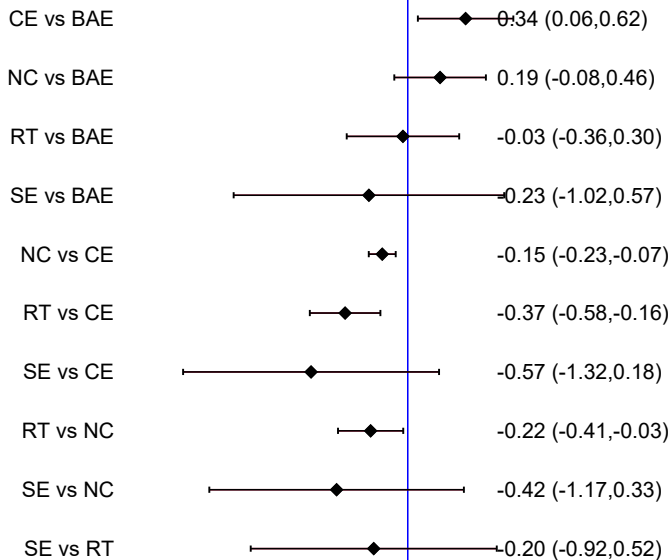

-1.3 -0.8 0.1 .6

Supplement: Supplementary file 1 [file Data_Sheet_1.ZIP › Supplementary material/Appendix 4.2-Pairwise comparisons of forest plots.pdf]
